# Isolated liver transplantation for treatment of liver failure secondary to intestinal failure

**DOI:** 10.1186/1824-7288-35-28

**Published:** 2009-09-15

**Authors:** Maria Immacolata Spagnuolo, Eliana Ruberto, Alfredo Guarino

**Affiliations:** 1 Department of Paediatrics, University Federico II of Naples, Via S. Pansini, 5, 80131 Naples, Italy

## Abstract

Intestinal Failure is a permanent loss of digestive and absorptive functions as a consequence of short bowel syndrome and/or other primary intestinal conditions. Patients with intestinal failure (IF) require long term parenteral nutrition to survive. The only alternative to parenteral nutrition is intestinal transplantation which still entails high mortality. Children with intestinal failure on parenteral nutrition may develop liver failure (LF), as a consequence of central line infections and other conditions. In children with both irreversible IF and LF a combined transplantation is generally considered. Despite low survival rate, combined liver/intestine transplantation is associated to better intestinal graft survival and lower incidence and severity of rejection compared to isolated small bowel transplantation. Recently, isolated liver transplantation was proposed in children with IF and LF. This procedure may have a higher survival probability compared to isolated intestinal transplant, it may allow progressive weaning from PN in children in whom the remnant intestine has the potential for adaptation and offer a timely solution in children for whom intestinal graft is not immediately available. This innovative approach may prove a better option compared to combined transplantation in both the short and long term

## Introduction

**Figure 1:**
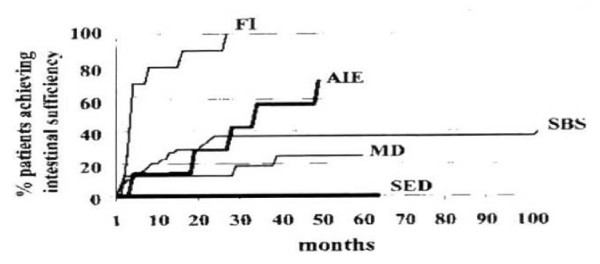
**Time-related gaining of intestinal sufficiency according to the cause**. For each group, the probability of being weaned from parental nutrition is reported as a time-related function. Each cause showed rates of intestinal sufficiency that were significantly different from all other causes (P < 0.00001).

Primary Intestinal Failure (IF) is defined as a long term or irreversible loss of intestinal functions. Intestinal functions include digestion and absorption of nutrients, hydroelectrolyte homeostasis and motility. The intestine is also the major site of microflora and of the of immune system. IF is the result of severe primary intestinal diseases or extraintestinal diseases. The former include the short bowel syndrome (SBS) and and other primary intestinal conditions whose onset is often in neonatal age [1-3]. The natural history of intestinal failure strongly depends on its primary etiology and the management and children with structural enterocyte effects have the worst outcome (figure 1) [4]. As a consequence of IF, Parenteral Nutrition (PN) is required to ensure survival and growth [3,5,6].

IF may be irreversible and require life-long total or supplemental parenteral nutrition or may be transient and allow a partial or total recovery of all or most of intestinal functions [3]. Intestinal adaptation, defined as the functional and structural changes of the remaining intestine, determines the chance to restore intestinal sufficiency. Generally when essential digestive functions are restored, PN is no larger needed

Although a life-saving option, PN is associated with a number of severe complications, including metabolic abnormalities, catheter-related sepsis, loss of vascular accesses and parenteral-associated cholestasis [3]. PN is associated with substantial costs, due to the high tech requirements and its long duration. The only alternative to PN is intestinal transplantation (ITx) [5-7]. Current immunosuppressive regimens have substantially improved the long term outcome and the international registry for ITx reports a rapid and progressive increase in the number of transplants performed as well as number of centers for ITx [7]. However, ITx still entails high mortality and substantial costs [8]and the decision to refer a child for ITx should be based on the best prognostic evaluation of each individual case as suggested by position paper with the indications to ITx was produced in 2001 [9]. However, most children remain on PN, either or not awaiting IT, and may eventually develop intestinal failure-associated liver disease (IFALD) [9-14]. As many as 40 to 50% of the children on long-term PN eventually develop abnormal liver function and 10% undergo liver failure [12]. The risk of cholestasis and liver failure was strongly reduced by the introduction of early treatment with UDCA [15,16]. However, IFALD is, still now and in major reference centers, a major complication of IF and a cause of fatal events. The intestinal transplant registry reports poor survival in children younger than 2 years because of the rapid progression of IFALD, while awaiting appropriate size-matched donor organs [17]. Combined liver and small bowel transplantation (LSBTx) is an established lifesaving treatment option for children with SBS and progressive IFALD [15], but is loaded with substantial mortality, problems in management and costs [18].

These evidences have forced the pediatric transplant community to reevaluate alternative options for infants with IF and progressive IFALD and in particular in children with SBS, the most frequent cause of IF. A feasible option is to perform Isolated Liver Transplantation (iLTx) in a selected group of patients with SBS and IFALD [18]. iLTx may be considered: 1, as a an emergency life saving option for children with end stage liver failure for which no intestinal graft is available; 2, for children who are expected to reach intestinal sufficiency and therefore would need liver but not intestinal transplant; 3, for children who are doing well on PN and therefore are not really candidates to intestinal transplantation.

## Discussion

The outcome of infants with SBS is associated with age-adjusted small bowel length and function [19-22], the rate of PN complications [12,20], and the presence of the ileocaecal valve (ICV) and colon [3,11]. Approximately 15-20% of children with post surgery SBS and PN for more than 4 weeks are eventually able to gain intestinal sufficiency within 2 years (figure 1).

Long-term complications of PN regimens are considered as major indications to ITx, and include: 1) progressive PN-associated liver disease defined as persistence of plasma direct bilirubin above 3 mg/dl for at least 4 months or irreversible liver disease with cirrhosis, 2) recurrent catheter-related sepsis, defined as at least 2 central line infections (CLIs) per year or 1 episode of fungal sepsis or 1 major septic complication (i.e. septic shock, multi-organ failure, metastatic abscess), 3) lack of central venous access, and specifically the irreversible loss of 2 of the four available standard access sites for infants and 3 of the 6 for older children [9].

In parallel, PN has also been an area of substantial improvement with an important reduction of major complications such as micronutrient deficiencies, loss of vascular accesses and central line infections [23]. The combined progresses in PN are likely to lead to an increased number of children with IF who will ultimately be able to be weaned from PN.

Pharmacologic or surgical approaches to increase bowel adaptation, such as Growth Hormone-or Epidermal Growth Factor administration or bowel lengthening procedures, have been described and may increase the rates of children weaned from PN [3,5,20,24-26]. Nevertheless, enteral nutrition remains the most important prognostic determinant for weaning from PN, as nutrients exert a direct trophic effect on enterocytes and selective nutrients such as lactoferrin may specifically induce enterocyte growth and differentiation [27]. Intestinal nutrients stimulate gastro-enteral secretions, promote hormonal factors, thereby enhancing gut adaptation [3,5,20,28].

Adaptation is hampered by IFALD, namely by a combination of impaired synthetic liver function, portal hypertension, bowel wall oedema, anorexia and portal enteropathy, all of which perpetuate PN dependence and increase the risk for recurrent central line infections.

Combined LSBTx is nowadays considered as the standard treatment option for IFALD. However, survival rate of children undergoing combined LSBTx is low, with 65% mortality rate at 6 months [20,29-33]. Somehow surprisingly compared to isolated ITx, combined transplantation is associated to better intestinal graft survival and lower incidence and severity of rejection [3,34,35]. In particular, there is a lower incidence and severity of acute intestinal graft rejection in recipients of combined transplantation than in recipients of isolated small bowel transplantation, probably as a consequence of immune tolerance induced by the liver.

Recently, isolated LTx has been introduced in the management of these patients. Initially performed as an emergency procedure in patients with end stage liver failure awaiting intestinal graft, isolated LTx may actually restore liver sufficiency ultimately allowing a later intestinal adaptation that may lead in selected patients to complete enteral autonomy [30,31].

The impact of isolated liver transplantation on either survival and quality of life of patients with intestinal failure and associated liver disease is encouraging [36-39]. Dell'Olio et al. in a recent paper reported that isolated LTx may be life saving for selected children with SBS and IFALD in whom the residual bowel has the potential for intestinal adaptation [40]. The authors proposed new criteria for isolated LTx in children with IFALD. In a series of 14 children receiving ILTx, 9 (64%) were alive after a median period of 35.6 months (range 21.2-86.8) of whom 8 were successfully weaned from parenteral nutrition after a median period of 15 months. Three year survival of a parallel cohort of children who received combined LSBTx between 1998 and 2005 was 48% [41]. In other words, if these patients would had received a combined LSBTx rather than an isolated LTx, during the same period of time, the majority of them would have died. This data raise the option of performing isolated LTx rather than combined LSBTX in children doing well on PN.

On the basis of their experience, Dell'Olio et al. proposed the following modifications to their own original criteria for isolated LTx to the following: 1. established IFALD (serum bilirubin >200 mmol/L, moderate/severe fibrosis, portal hypertension); 2. at least 50 cm functional small bowel remaining intact in the absence of ICV or 30 cm with ICV; 3. at least 50% of the estimated daily energy requirement was tolerated as enteral feeds, for at least 4 weeks before the development of liver disease and was associated with an increase in weight; 4. children with dilated and dysmotile bowel who had minimal line infections (<6 in 12 months).

It is clear that length of residual bowel play a significant role in predicting whether intestinal failure may be reversible. However function is more important than length oral may be assessed by gradually decreasing parenteral nutrition and increasing oral intake in parallel. There is no scheme to do that and the task is not easily achieved. However intestinal function tests may be used to non invasively monitor intestinal function, thereby providing information on time related adaptation and guiding progressive weaning. A list of intestinal function test and their interpretation is provided in table 1. [42]

**Table 1:** Non-invasive tests for intestinal and pancreatic digestive-absorptive functions and for intestinal inflammation.

**Test**	**Normal values**	**Implication**	**Reference**
α1-antitrypsin concentration	< 0.9 mg/g	increased intestinal permeability/protein loss	Catassi C et al. J Pediatr 1986;109:500-502

Steatocrit	<2.5% (older than 2 years)	fecal fat loss	Guarino A et al. J Pediatr Gastroenterol Nutr1992;14:268-274

Fecal reducing substances	absent	carbohydrate malabsorption	Lindquist BL et al. Arch Dis Child 1976;51:319-321

Elastase concentration	> 200 ug/g stool	exocrine pancreatic dysfunction	Carroccio A et al. Gut 1998;43:558-563

Chymotrypsin concentration	> 7.5 U/g > 375 U/24 h	exocrine pancreatic dysfunction	Carroccio A et al. Gastroenterology 1997;112:1839-1844

Fecal occult blood	absent	fecal blood loss, distal intestinal inflammation	Fine KD. N Engl J Med 1996;334:1163-1167

Calprotectin concentration	100 ug/g	intestinal inflammation	Fagerberg UL et al. J Pediatr Gastroenterol Nutr 2003;37:468-72

Fecal leukocytes	< 5/microscopic field	colonic inflammation	Harris JC et al. 1972;76:697-703

Nitric oxide in rectal dyalisate	< 5 uM of NO_2_^-^/NO_3_^-^	rectal inflammation	Berni Canani R et al. Am J Gastroenterol 2002;97:1574-1576

Dual sugar (cellobiose/mannitol) absorption test	Urine excretion ratio: 0.010+0.018	Increased intestinal permeability	Catassi C, et al. J. Pediatr Gastro Nutr 2008;46:41-47

Xylose oral load	25 mg %	Absorptive surface	Craig RM, Ehrenpreis ED J Clin Gastroenterol 1999; 29:143-50

Iron absorption test	Based on percentile reference		De Vizia et al. J. Pediatri Gastroentrol Nutr. 1992;14-21-6

Intestinal digestive-absorptive processes may improve following isolated LTx because portal hypertension and bowel oedema could have contributed to intolerance to enteral feeds in the pretransplant phase [18,43].

In patients with severe motility impairment or nutrient malabsorption, full adaptation is unlikely, and the chance of weaning is low. In these patients either long term PN or combined LSBTX should be considered. Finally a complications is the relatively high risk of food allergy, that complicates evaluation of tolerance

The success of isolated LTx is limited by frequent postoperative complications The rates of infectious, vascular, biliary, and surgical complications are higher compared with patients undergoing liver transplants for other indications [18].

A potential problem of patients with intestinal failure receiving isolated LTx could be antirejection drugs malbsorption but it has been reported that therapeutic levels of these drugs may be achieved in these patients in the postoperative period [44].

A major problem of iLT in patients with IF is its cost. The high rates of post-operative complications contribute to high costs, as do extended hospital and intensive care unit stay. PN administration also increases the already heavy post-transplantation charges. These costs need to be balanced against the cost of complications and hospitalizations for patients on the waiting list for combined LSBTX. As these patients may ultimately skip intestinal transplantation, improving long-term outcomes and survival, the cost on the front end may well be justified [43,45].

## Conclusion

In IF therapeutic options are complex, and not well defined and yet they are potentially crucial for patient survival and associated with high costs. The therapeutic strategy impact on quality of life is correspondingly high.

Long term data on the history of patients with IF and LF treated with different approaches should be obtained to better define when isolated LTx vs combined transplantation should be used for IFALD,. This is hampered by the low number of patients, their scattered clinical conditions and the rapid progresses that are being made in various areas of IF including clinical nutrition, growth factors, microflora modulation and central line management. On the other side, there are substantial progresses in either surgery and immune suppression. These progresses are likely to open new hopes for children that only few years ago in rich Countries - and now also in less rich Countries- are admitted for IF.

## Competing interests

The authors declare that they have no competing interests.

## Authors' contributions

MIS carried out the revision of the literature and drafted the manuscript, ER participated in the sequence alignment, AG participated in its design and coordination. All the authors read and approved the final manuscript.
